# Automatic Reappraisal-Based Implementation Intention Produces Early and Sustainable Emotion Regulation Effects: Event-Related Potential Evidence

**DOI:** 10.3389/fnbeh.2020.00089

**Published:** 2020-07-14

**Authors:** Shengdong Chen, Kang Yu, Jiemin Yang, Jiajin Yuan

**Affiliations:** ^1^The Affect Cognition and Regulation Laboratory (ACRLAB), Key Laboratory of Cognition and Personality of Ministry of Education (SWU), Faculty of Psychology, Southwest University, Chongqing, China; ^2^Institute of Brain and Psychological Sciences, Sichuan Normal University, Chengdu, China

**Keywords:** cognitive reappraisal, implementation intention, emotional intensity, event-related potentials, late positive potential

## Abstract

Implementation intention has proven effective in regulating intense emotions but is found to be difficult when instructed regulation is used. Here, we aim to test whether automatic reappraisal-based implementation intention (RII) downregulates intense negative emotion more efficiently than controlled reappraisal (CR) using a two-phase event-related potential (ERP) experiment. In the regulation phase, both RII and CR decreased subjective experiences of negative emotion relative to passive watching, irrespective of emotional intensity. Moreover, RII reduced the central–parietal late positive potential (LPP) amplitudes for both intensities in the 300–1,700-ms epoch after picture onset, whereas CR reduced LPP amplitudes just in the 500–700-ms interval. Moreover, the application of RII but not CR produced a reliable long-term LPP attenuation compared to passive watching in the unexpected re-exposure phase. These findings demonstrate that reappraisal-based implementation intention yields an earlier and more sustainable emotion regulatory effects than controlled reappraisal.

## Introduction

Cognitive reappraisal involves construing an emotional situation in nonemotional terms during emotion regulation (Sheppes and Gross, 2011). This self-regulation strategy has been established to effectively downregulate subjective emotional experiences (Ochsner et al., 2004; Ray et al., 2010), emotion-expressive behavior (Gross, 1998), and amygdala activation (Chen et al., 2017). However, recent studies indicate that the controlled reappraisal (CR) initiated by explicit and conscious instructions is less effective and more effortful in high than in low emotional intensity (Sheppes et al., 2014; Shafir et al., 2015). For example, CR resulted in weaker modulation of self-reported negative experience compared with distraction (Shafir et al., 2015) or attentional deployment (Sheppes et al., 2014) even though CR was as effective as these strategies in downregulating low-intensity negative emotions.

**Figure 1:**
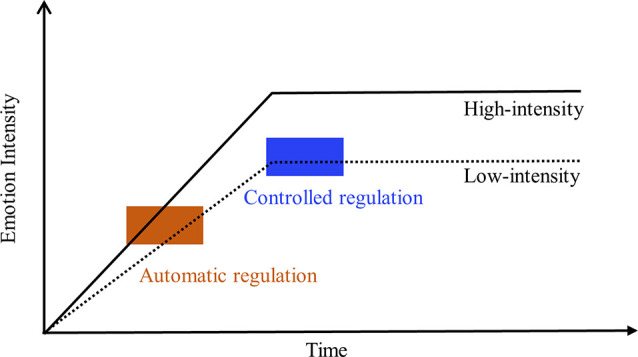
The hypothesis of emotion-regulatory speed and effect of automatic regulation vs. controlled regulation in downregulating low- and high-intensity emotion. The size of the rectangle reflects the regulatory effects of automatic regulation (khaki) and controlled regulation (blue) on low- or high-intensity emotional responses.

Previous studies have suggested that automatic cognitive processes operate earlier than controlled cognitive processes (Beck and Clark, 1997; Hahne and Friederici, 1999). Consistently, increasing evidence shows that automatic self-regulation is activated quickly and resists to ego-depletion efficiently (Webb and Sheeran, 2003; Fitzsimons and Bargh, 2004; Gallo et al., 2009). Given that one self-regulation strategy can operate in controlled or automatic forms (Gross, 1998; Braunstein et al., 2017), automatic emotion regulation may interrupt the development of an emotional impulse earlier than controlled emotion regulation. According to the process model of emotion regulation (Gross, 1998), the earlier an emotional impulse is interrupted, the less experiential and physiological emotional responses are generated (Figure 1). Consequently, the emotional regulatory effects of automatic cognitive reappraisal should be more prominent than CR, particularly during intense emotional situations. Additionally, previous research indicates that automatic cognitive processing leads to long-term retention of associated skills (Schneider and Chein, 2003), suggesting that automatic emotion regulation may provide long-term emotion regulation effects.

However, no research has attempted to examine whether the short- and long-term regulatory effects of automatic reappraisal are impacted by emotional intensity and the underlying temporal mechanisms. Given that event-related potentials (ERPs) have been widely used as the temporally fine-grained indices of the effects of reappraisal, we designed an ERP study including regulation and re-exposure phases to examine the short- and long-term regulatory effects of automatic reappraisal, respectively. Specifically, we collected self-report ratings of valence and arousal and used the centro-parietal late positive potential (LPP) as an ERP index, since LPP has been suggested to be sensitive to both emotional intensity (Shafir et al., 2016) and cognitive reappraisal process (Hajcak et al., 2006, 2010). The centro-parietal LPP starts 300 ms after stimulus onset, showing enhanced amplitudes as the processing of emotional intensity increases (Hajcak et al., 2009). Importantly, 300–1,700 ms of centro-parietal LPP is typically used to show the regulatory effects of cognitive reappraisal (Foti and Hajcak, 2008; Thiruchselvam et al., 2011; Shafir et al., 2015; Qi et al., 2017). Besides, we used the frontal LPP as an objective index of cognitive effort, since previous ERP studies observed an enhanced LPP at frontal sites during implementing controlled reappraisal relative to passive watching (Bernat et al., 2011; Moser et al., 2014; Shafir et al., 2015).

Furthermore, we used the implementation intention paradigm to initiate automatic reappraisal. Implementation intentions refer to the if–then plans that specify when, where, and how individuals will strive towards particular goals (Gallo et al., 2009). Implementation intention paradigm has been suggested to be effective in automatically reducing subjective and physiological responses to negative emotional stimuli (see meta-analysis by Webb et al., 2012), without taxing self-regulatory resources (Gallo and Gollwitzer, 2007) or increasing conscious perception on the regulatory processing (Gallo et al., 2012). For example, Gallo et al. (2012) found that participants who formed reappraisal-based implementation intention (RII, e.g., “if I see blood, then I will take a perspective of a physician!”) rated disgusting pictures as less unpleasant than participants in the watching or mere goal-intention groups, without consciously perceiving themselves as being more successful. More importantly, indirect evidence indicates that automatic emotion regulation supported by implementation intention results in earlier attenuation of neural activity than controlled emotion regulation. Gallo et al. (2009) found that participants who formed an implementation intention showed a lower positivity in the P1 (60–150 ms) when viewing threatening pictures as compared to participants given a goal intention and to no-goal control participants. In contrast, the regulatory effects of CR generally appear during the LPP phase (>300 ms; Foti and Hajcak, 2008; MacNamara et al., 2009; Moser et al., 2014; Qi et al., 2016).

Moreover, because we were interested in differences between automatic and controlled forms of reappraisal, we focused on one type of cognitive reappraisal (i.e., perspective-taking reappraisal) to avoid the confounding of types of reappraisal. Perspective-taking reappraisal askes participants to alter the impact of the emotional stimulus by adopting a third-person perspective, which has been suggested to have a larger effect size in modulating emotional outcomes than the other types of cognitive reappraisal (e.g., reappraising emotional response or emotional stimulus; see Webb et al., 2012 for more details). In terms of how a stimulus is appraised, perspective-taking reappraisal is a case of detached reappraisal (also called as self-focused) that reinterprets one’s subjective relationship to emotional events in a detached and unemotional way (Qi et al., 2017). Consistent with the meta-analysis by Webb et al. (2012), our recent ERP study also found that detached reappraisal supported by implementation intention led to lower physiological responses to disgusting stimuli than positive reappraisal supported by implementation intention that requires a reinterpretation of an emotionally charged situation in a constructive manner (Ma et al., 2019).

The regulation phase of this study guided participants in the passive watching, RII, and CR groups passively view, automatically regulate, and controllably regulate disgust images, respectively. In the regulation phase, we first hypothesized that RII would result in earlier and enhanced attenuation of the centro-parietal LPP than CR, which in turn was expected to be more effective during intense emotional situations. Behaviorally, we hypothesized that relative to the passive watching (control condition), RII would result in less negative experience irrespective of low or high intensity, whereas CR would only result in less negative experience in low (but not high)-intensity condition. Given the automatic characteristics of RII (Gallo and Gollwitzer, 2007; Gallo et al., 2009), we also hypothesized that RII relative to CR would require less cognitive control, as indicated by stronger attenuation of the frontal LPP. The re-exposure phase began 20 min later, during which all groups passively viewed the same pictures. The re-exposure task was designed to explore whether RII had long-term effects on self-reported arousal and the central–parietal LPP.

Moreover, we chose disgust as the target emotion because it reliably induces both enhanced subjective emotional ratings and LPP responses (Schienle et al., 2008; Wheaton et al., 2013). We restricted this study to women who are more susceptible to negative emotions (Yuan et al., 2009) and generally show higher disgust sensitivity than men (Schienle et al., 2002, 2005; Curtis et al., 2004).

## Materials and Methods

### Participants

Seventy-five (25 participants per group) healthy, right-handed female undergraduates (*M*_age_ = 19.85, *SD* = 1.39) participated in this study, with normal or corrected-to-normal vision. Because this experiment mainly focused on the LPP responses, we determined the sample size based on the recent majority of ERP studies in the field of cognitive reappraisal (e.g., Shafir et al., 2016; Qi et al., 2017). Written informed consent was obtained before the experiment. This study was approved by the local ethical committee of the Faculty of Psychology at Southwest University.

We also examined the group differences (watching, RII, and CR) in emotional states, emotion-related traits, and ages using one-way between-subjects ANOVAs. Results demonstrated no significant group differences (*p*s > 0.05) in the scores of positive/negative affect (PANAS, Watson et al., 1988), Spielberg State Anxiety Inventory, the Spielberg Trait Anxiety Scale (Spielberger, 1970), and the ages, suggesting that the three groups have no significant difference in the emotional baseline.

### Materials and Presentation

In order to find sufficient numbers of low- and high-intensity disgust pictures, we first collected pictures from both the International Affective Picture System (IAPS, Lang et al., 1999) and the Chinese Affective Picture System (CAPS, Lu et al., 2005). Specifically, two authors working in the domain of affective neuroscience for at least 2 years first collected the low- (e.g., slight cuts on hands) and high-intensity (e.g., severe facial burns) disgust pictures based on subjective experiences.

Furthermore, because of the two different sources (i.e., IAPS and CAPS) used for picture selection and the potential impact of cultural difference on ratings of IAPS pictures (Huang and Luo, 2004), we re-rated the collected pictures by an independent sample. Fifteen psychology graduate or doctoral students assessed the valence (1 = very unpleasant; 9 = highly pleasant) and arousal (1 = low; 9 = high) scores, and the degree they felt sadness, fear, joy, anger, and disgust on scales (1 = little; 9 = very) of each disgust picture presented in a randomized order. Results revealed a significant interaction effect between the emotional intensity and the emotion degree ratings, *F*_(4,472)_ = 115.69, *p* < 0.001, $\eta_{\text{p}}^{2}$ = 0.49. *Post hoc* Bonferroni tests showed that disgust (low/high, *M* = 5.55/7.21) was the most prevalent emotion for both low- and high-intensity pictures in comparison with sadness (low/high, *M* = 3.84/4.5, *p* < 0.001), anger (low/high, *M* = 3.49/4.23, *p* < 0.001), happiness (low/high, *M* = 2.27/1.73, *p* < 0.001), and fear (low/high, *M* = 3.65/5.93, *p* < 0.001), and that for each emotion rating, the differences between high and low pictures were significant (*p*s < 0.001). These findings suggest that the picture set we offered here can elicit low- and high-intensity disgust effectively.

The picture set comprised 150 pictures, including 60 low-intensity and 60 high-intensity disgust pictures with low valence (low/high, *M* = 2.86/1.87, *SD* = 0.47/0.36) and high arousal (low/high, *M* = 5.72/7.46, *SD* = 0.79/0.64) ratings, and 30 neutral pictures with medium valence (*M* = 5.12, *SD* = 0.52) and low arousal (*M* = 3.39, *SD* = 0.66) rating. Thirty low- and 30 high-intensity disgust pictures and 30 neutral pictures were selected from the picture set and were used in this study. These pictures were presented on a color monitor using E-prime 2 stimulus presentation software. Viewing distance was held constant at ~150 cm, and both horizontal and vertical visual angles were kept below 6°.

### Design and Procedure

This experiment used a 3 × 3 mixed design with the instruction type (watching, RII, and CR) as a between factor and emotional-intensity category (high, low, and neutral) as a within factor. Upon arrival, participants completed informed consent. Participants were then seated in a quiet room and completed emotion-related questionnaires. With these preparations completed, we began the two-phase ERP study.

In the following regulation phase, each group first underwent a 12-trial (four trials per picture type) practice phase. During this phase, participants were required to speak out how they implemented their instructions and were corrected as needed. The RII group received the following instructions to form an implementation intention: “I will not get disgusted! And if I see blood, then I will take a perspective of a physician!” Participants were not given a specific time (~1 min) to form their implementation intentions but were asked to read and repeat the instructions very carefully. Passive watching instructions involve paying close attention to the pictures and letting natural thoughts and feelings to arise. Controlled reappraisal instructions involved changing their perspective to decrease emotional reactivity to disgust pictures, for example, by assuming the perspective of a medical professional watching an instructional presentation. The experimenter explained how to use SAM to participants after giving instructions. Each group of participants reported that they understood and were familiar with the instructions and SAM when finishing the 12 practice trails.

The formal ERP task began after the practice stage. The watching and RII groups received the same instructions of passively watching images, and the CR group received the explicit instructions of cognitive reappraisal. RII group received no further emotion regulatory instruction. After receiving the instruction, participants started the picture presentations by pressing the “S” key. Each regulation or control task consisted of 30 low-intensity, 30 high-intensity negative, and 30 neutral pictures. Within each group and for each participant, the pictures were presented in a randomized manner. Each trial began with a fixation cross for 2–4 s, followed by a picture for 4 s. After the offset of each picture, participants rated their level of emotional valence and arousal using SAM.

After ~20-min resting, an unexpected re-exposure task was delivered where subjects were presented with the images from the earlier regulation task. For all of three experimental groups, the re-exposure task simply instructed participants to attend to each image and to report their emotional experiences naturally.

### EEG Recording and Analysis

We recorded electroencephalogram (EEG) at 500 Hz using an elastic cap (Brain Products) with 64 sensors according to the extended 10-20 system, with two additional mastoid electrodes and a ground electrode on the medial frontal aspect. We used the electrode FCz as an online reference and kept impedance below 5 kω. Raw EEG data were amplified with a 0.01–100 Hz band-pass and were filtered with a notch filter at 50 Hz.

Offline signal processing was carried out using EEGLAB (Delorme and Makeig, 2004). During the offline analysis, we first downsampled the EEG signal at 250 Hz and performed bandpass filter (0.01–40 Hz). We then removed nonbrain electrodes, rejected artifactual channels by the clean_rawdata plugin in EEGLAB, rereferenced the EEG data to the average activity of the left and right mastoids, and rejected epochs with nonstereotyped artifacts. We removed eye-movement artifacts using independent component analysis (ICA) approach. To improve the decomposition, the ICA was performed on the bandpass-filtered (1–40 Hz) raw data (excluding bad channels; Groppe et al., 2009; Winkler et al., 2015). The demixing matrix obtained at 1 Hz data was then applied to the 0.01 Hz filtered data. Eye-movement-related ICA components were marked by visual inspection and finally removed from the continuous EEG data.

For LPP analysis, the continuous EEG was epoched into segments. Baseline correction was then performed by subtracting the mean of a 200-ms prepicture period from the entire duration of picture presentation (4,000 ms). The centro-parietal LPP was measured as the average activity of CPz and Pz, where it is frequently observed (Hajcak et al., 2010; Shafir et al., 2015; Shafir and Sheppes, 2018). In order to better test the time points at which two forms of reappraisal modulated the centro-parietal LPP, the period (300–1,700 ms) of centro-parietal LPP was divided into seven equal 200-ms time segments (300–500, 500–700, 700–900, 900–1,100, 1,100–1,300, 1,300–1,500, and 1,500–1,700 ms). The method of dividing centro-parietal LPP into small time segments (i.e., 200 ms) is frequently used in previous ERP studies focusing the timing effects of cognitive reappraisal (e.g., Thiruchselvam et al., 2011; Paul et al., 2013; Shafir et al., 2015; Qi et al., 2017). Following Moser et al. (2014) and Shafir et al. (2015), the frontal LPP was measured as the average activity of FC1, FC2, and FCz between 700 and 1,100 ms following picture onset.

## Results

### Behavioral Measure of the Negative Experience

**Figure 2:**
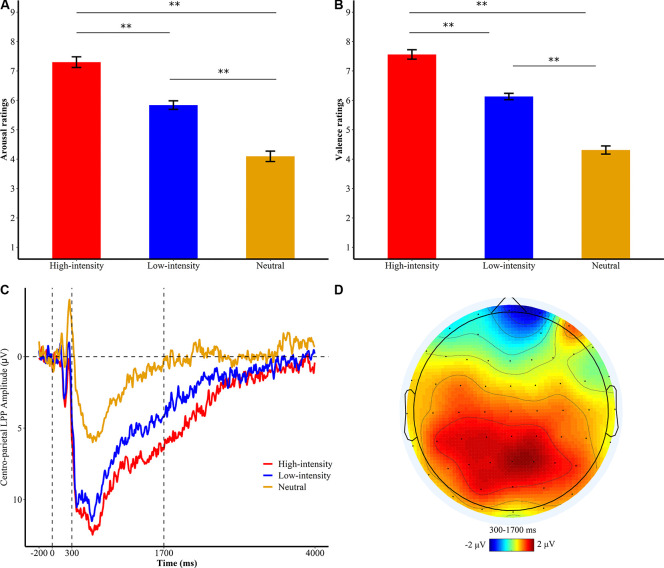
Subjective ratings of **(A)** emotional arousal and **(B)** valence of the passive watching group. **p < 0.001; bars represent standard error. **(C)** Centro-parietal late positive potential (LPP) amplitudes for different levels of emotional intensities of the watching group during the first exposure phase. Waveforms are averaged across CPz and Pz electrodes. The *x*-axis runs from the beginning of the baseline (−200 ms before picture onset) to the end of the picture presentation (4,000 ms). **(D)** Topographical distribution of the difference wave of high intensity minus neutral in the watching group.

For subjective ratings of valence or arousal, we conducted a 3 × 3 ANOVA with emotional intensity (high, low, and neutral) as a repeated-measures factor and instruction type (RII, CR, watching) as a between-participants factor to examine the effects of emotional intensity and to test the efficacy of RII and reappraisal in low and high emotional intensities. As expected, we found that the main effects of emotional intensity were significant for both arousal and valence ratings [arousal/valence, *F*_(2,144)_ = 360.78/374.91, *p*s < 0.001, $\eta_{\text{p}}^{2}$ = 0.83/0.84). Bonferroni planned comparisons showed that high-intensity pictures (arousal/valence, *M* = 6.62/6.95, *SD* = 0.11/0.10) were experienced as more negative than low-intensity pictures (*M* = 5.39/5.76, *SD* = 0.09/0.06, *p*s < 0.001) and neutral pictures (*M* = 3.74/4.24, *SD* = 0.11/0.08, *p*s < 0.001). Low-intensity pictures were also experienced as more negative than neutral pictures (*p*s < 0.001), suggesting a successful experimental manipulation of high- and low-intensity emotion (Figures 2A,B).

**Figure 3:**
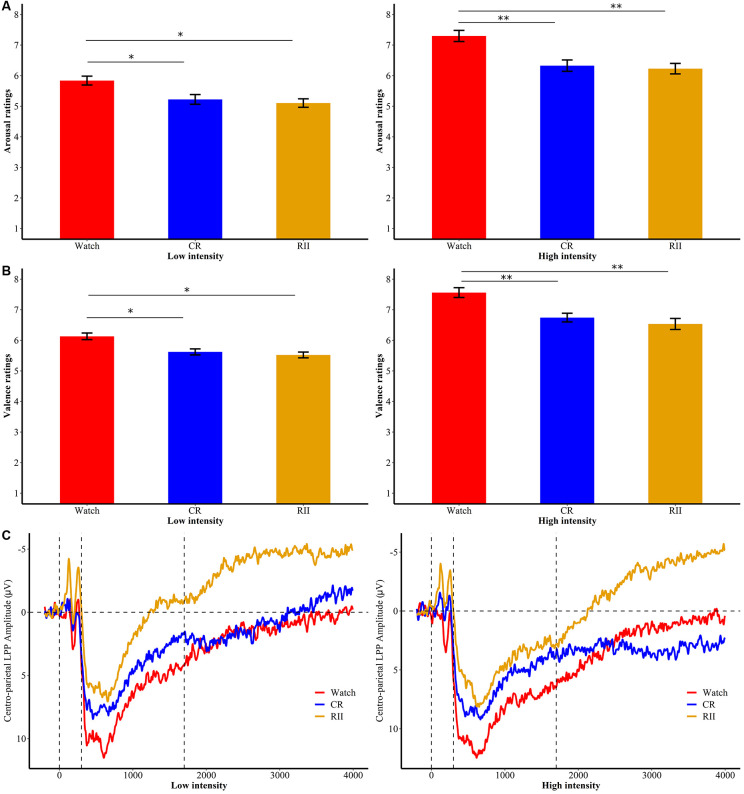
Subjective ratings of emotional **(A)** arousal and **(B)** valence for RII, CR, and watch groups in low- and high-intensities of the first exposure. *p < 0.05; **p < 0.001; bars represent standard error. RII, reappraisal by implementation intention; CR, controlled reappraisal. **(C)** Centro-parietal LPP amplitudes for RII, CR, and watch groups in high and low emotional intensities of the first exposure. Waveforms are averaged across CPz and Pz electrodes. The *x*-axis runs from the beginning of the baseline (−200 ms before picture onset) to the end of the picture presentation (4,000 ms).

Furthermore, we found no significant interaction between Emotional-Intensity and Instruction-Type on arousal ratings (*F*_(4,144)_ = 1.23, *p* = 0.30, $\eta_{\text{p}}^{2}$ = 0.033), but a significant main effect of Instruction-Type (*F*_(2,72)_ = 8.45, *p* < 0.001, $\eta_{\text{p}}^{2}$ = 0.19). Follow-up Bonferroni comparisons showed that RII (*M* = 4.94, *SD* = 0.77) and CR (*M* = 5.06, *SD* = 0.77) were both effective in reducing arousal ratings compared with the watching group (*M* = 5.74, *SD* = 0.77), yet with no difference between these two regulatory groups (*p* > 0.1; Figure 3A). Moreover, we found a significant interaction between Emotional-Intensity and Instruction-Type on valence ratings, *F*_(4,144)_ = 4.20, *p* = 0.003, $\eta_{\text{p}}^{2}$ = 0.105. Follow-up Bonferroni comparisons showed that RII (low/high, *M* = 5.52/6.54, *SD* = 0.60/0.97, *p*s < 0.001) and CR groups (*M* = 5.62/6.74, *SD* = 0.53/0.77, *p*s = 0.004) significantly reduced unpleasantness of both low- and high-intensity disgust pictures compared with the watching group (*M* = 6.13/7.56, *SD* = 0.58/0.86), also with no significant difference between RII and CR (*p*s > 0.1; Figure 3B). No significant group differences were found in the valence ratings of neutral pictures (*p*s > 0.5).

**Figure 4:**
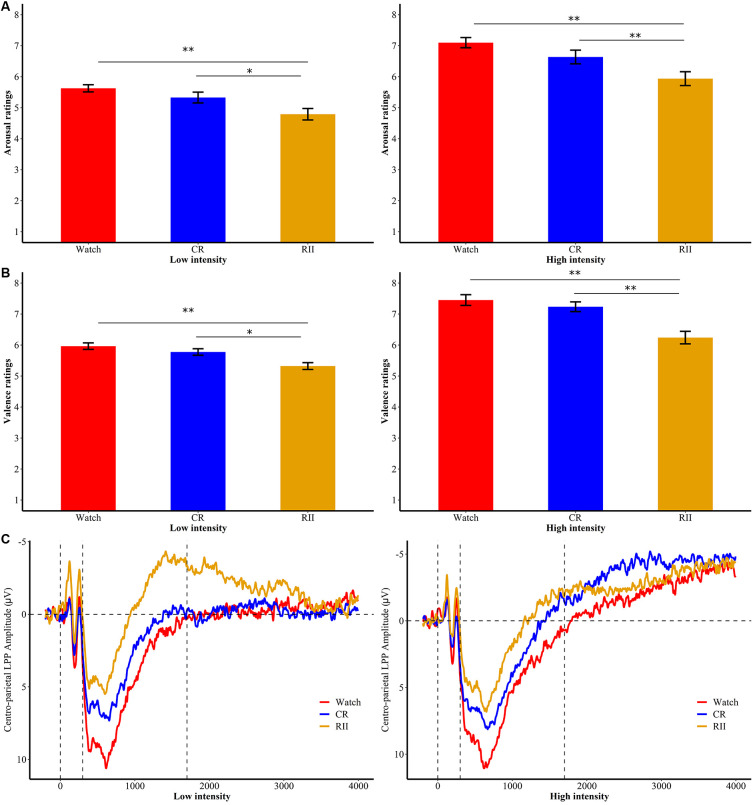
Subjective ratings of **(A)** emotional arousal and **(B)** valence for RII, CR, and watch groups in high and low emotional intensities of the re-exposure task. *p < 0.05; **p < 0.001; bars represent standard error. RII, reappraisal by implementation intention; CR, controlled reappraisal. **(C)** Centro-parietal LPP amplitudes for RII, CR, and watch groups in high and low emotional intensities of the re-exposure task. Waveforms are averaged across CPz and Pz electrodes. The *x*-axis runs from the beginning of the baseline (−200 ms before picture onset) to the end of the picture presentation (4,000 ms).

In the re-exposure task, we examined whether self-reported ratings of valence and arousal varied as a function of instruction history (RII, CR, and watching) and emotional intensity (high, low, and neutral). For arousal ratings, we found no significant interaction effect between instruction history and emotional intensity (*F*_(4,144)_ = 0.68, *p* = 0.61), but significant main effects of instruction history and emotional intensity (*F*_(2,72)/_*F*_(2,144)_ = 7.93/290.87, *p*s ≤ 0.001, $\eta_{\text{p}}^{2}$ = 0.18/0.80). Bonferroni planned comparisons showed that RII (*M* = 4.64, *SD* = 0.83) reduced arousal ratings compared with both CR (*M* = 5.16, *SD* = 0.83, *p* = 0.068) and watching (*M* = 5.54, *SD* = 0.83 *p* < 0.001) groups (Figure 4A). For valence ratings, we found a significant interaction effect between instruction history and emotional intensity, *F*_(4,144)_ = 4.23, *p* = 0.003, $\eta_{\text{p}}^{2}$ = 0.11 (Figure 4B). Follow-up Bonferroni comparisons showed that for both low- and high-intensity disgust pictures (but not neutral pictures, *p*s > 0.5), RII (low/high, *M* = 5.32/6.24, *SD* = 0.59/1.08) significantly reduced valence ratings compared with both CR (*M* = 5.77/7.24, *SD* = 0.58/0.83, *p*s < 0.02) and watching (*M* = 5.96/7.45, *SD* = 0.56/0.94, *p*s ≤ 0.001) groups.

### Neural Measures of Regulatory Modulation and Effort: Centro-parietal and Frontal-LPP Analysis

To test whether the neural modulation differences between RII and CR differ across low- and high-intensity levels, we employed a 7 × 3 × 3 ANOVA with time window (time segment) and emotional intensity (high, low, and neutral) as within-participants factors and instruction type (RII, CR, and watching) as a between-participants factor. Consistent with results of subjective ratings, the main effect of emotional intensity was also significant on LPP responses, *F*_(2,864)_ = 44.62, *p* < 0.001, $\eta_{\text{p}}^{2}$ = 0.383. High-intensity pictures (*M* = 6.54, *SD* = 5.83) elicited larger LPP amplitudes than low-intensity pictures (*M* = 4.84, *SD* = 5.52, *p* = 0.026) and neutral pictures (*M* = −0.08, *SD* = 7.80, *p* < 0.001), and low-intensity pictures elicited a larger LPP amplitude than neutral pictures (*p* < 0.001; Figure 2C).

Importantly, we found a significant time window × emotional intensity × instruction type interaction (*F*_(24,864)_ = 1.89, *p* = 0.006, $\eta_{\text{p}}^{2}$ = 0.05), and a significant main effect of instruction type, *F*_(2,72)_ = 5.36, *p* = 0.007, $\eta_{\text{p}}^{2}$ = 0.13. In order to examine whether RII would decrease centro-LPP amplitudes earlier than CR and whether its effects would be impacted by emotional intensity, we then performed two-way ANOVAs in each time segment with emotional intensity (high, low) as a within-participants factor and instruction type (RII, reappraisal, and watching) as a between-participants factor. We observed no significant interaction effects between instruction type and emotional intensity across the seven time segments, *F*s_(2,72)_ < 0.96, *p*s > 0.1 but significant main effects of instruction type across the first six time segments, *F*s_(2,72)_ > 3.24, *p*s < 0.05. Because of multiple statistical comparisons for the main effects of instruction type, we applied Finner’s procedure to control type I error (Finner, 1993), which is a stepwise method to control the familywise error rate that has more power than the classical Bonferroni correction. Planned comparisons showed that RII led to lower centro-LPP amplitudes relative to the watching group during all the seven time segments (300–1,700 ms; *p*s < 0.04; Figure 3C). In contrast, CR led to significantly lower centro-LPP amplitudes than watching only during the second segment (500–700 ms, *p*s = 0.03; Figure 3C). Moreover, the significant results of RII across seven time segments, when corrected for multiple comparisons, were still significant, whereas the significant result of CR disappeared. The *t* values, *p* values, means, and standard deviations of LPP of three groups across the seven time segments are presented in Tables s 1, 2. No other significant difference was observed between the three groups.

**Table 1:** Means (standard deviations) and pair-wise comparisons of the central–parietal late positive potential (LPP) between watch and controlled reappraisal (CR) groups in each 200-ms interval of the 300–1,700-ms time epoch.

Time (ms)	Watch	CR	*t*-value	*p*-value	Finner’s *p*-value
300–500	9.46 (5.47)	7.13 (5.81)	1.46	0.15	0.38
500–700	11.15 (4.79)	8.16 (4.91)	2.17	0.03	0.19
700–900	9.46 (5.21)	7.33 (4.87)	1.49	0.14	0.38
900–1,100	7.38 (5.09)	5.27 (4.73)	1.52	0.13	0.38
1,100–1,300	6.45 (5.63)	4.40 (5.09)	1.35	0.18	0.38
1,300–1,500	6.13 (5.88)	3.81 (5.20)	1.48	0.14	0.38
1,500–1,700	5.48 (6.14)	2.96 (5.84)	1.49	0.14	0.38

**Table 2:** Means (standard deviations) and pair-wise comparisons of the central–parietal late positive potential (LPP) between watch and reappraisal-based implementation intention (RII) groups in each 200-ms interval of the 300–1,700-ms time epoch.

Time (ms)	Watch	RII	*t-*value	*p*-value	Finner’s *p*-value
300–500	9.46 (5.47)	4.58 (6.41)	2.89	0.006	0.0277
500–700	11.15 (4.79)	6.80 (5.37)	3.02	0.004	0.0277
700–900	9.46 (5.21)	5.70 (5.58)	2.46	0.018	0.0369
900–1,100	7.38 (5.09)	3.41 (6.21)	2.47	0.017	0.0369
1,100–1,300	6.45 (5.63)	1.92 (7.07)	2.50	0.016	0.0369
1,300–1,500	6.13 (5.88)	1.25 (7.86)	2.49	0.016	0.0369
1,500–1,700	5.48 (6.14)	0.93 (8.85)	2.12	0.04	0.040

Concerning the centro-parietal LPP during the unexpected re-exposure task, we also conducted a 7 × 2 × 3 ANOVA with time window (seven time segments) and emotional intensity (high, low, and neutral) as within-participants factors and instruction history (RII, CR, and watching) as a between-participants factor. Results yielded no interaction effect with instruction history (*F*s ≤ 1.1). However, we found a marginally significant main effect of instruction history, *F*_(2,72)_ = 3.09, *p* = 0.052, $\eta_{\text{p}}^{2}$ = 0.079. Planned comparisons showed that RII (*M* = −0.27, *SD* = 5.33, *p* = 0.051), but not CR (*M* = 2.18, *SD* = 5.33, *p* = 1.0), led to lower centro-LPP amplitudes than watching group (*M* = 3.41, *SD* = 5.33; Figure 4C). No other significant or marginal differences between the three groups were observed (*p*s > 0.1).

**Figure 5:**
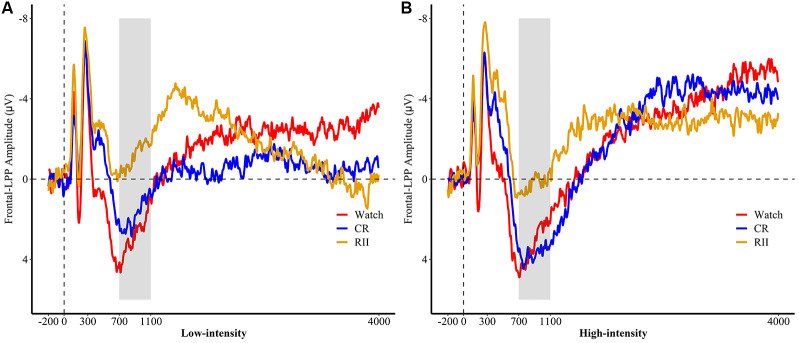
Picture-locked event-related potentials (ERP) in **(A)** low and **(B)** high emotional intensities for the re-exposure task, pooled at fronto-central sites (FC1, FC2, FCz). Picture onset is identified with a dotted line on *x*-axis. The *x*-axis runs from the beginning of the baseline (−200 ms before picture onset) to the end of the picture presentation (4,000 ms). RII, reappraisal by implementation intention; CR, controlled reappraisal.

We then conducted analyses to estimate the differential requirement of RII and controlled reappraisal for cognitive effort measured by frontal LPP. For both the first exposure and re-exposure tasks, we employed a 3 × 3 ANOVA with emotional intensity (high, low, and neutral) as a within-participants factor and instruction type or instruction history (RII, CR, and watching) as a between-participants factor. We found neither significant interaction effects between emotional intensity and instruction type/instruction history (*F*s ≤ 1.0, *p*s > 0.1) nor for the main effect of instruction type (*F*_(2,72)_ = 1.24, *p* = 0.29). However, we found a marginally significant main effect of instruction history, *F*_(2,72)_ = 2.93, *p* = 0.06, $\eta_{\text{p}}^{2}$ = 0.075. Planned comparisons showed that pictures with RII-history (*M* = −1.86, *SD* = 5.91, *p* = 0.034) but not with CR-history (*M* = 1.52, *SD* = 5.91, *p* = 0.89) elicited a lower amplitude of frontal LPP than pictures with Watching-history (*M* = 1.75, *SD* = 5.91; Figures 5A,B).

## Discussion

Finding more effective emotion regulation strategies is a continuing concern within the field of emotion. Although cognitive reappraisal has been suggested to be powerful in downregulating negative emotion, its implementation process (Shafir et al., 2015) and use frequency (Sheppes et al., 2011) have been suggested to be impacted by emotional intensity. The present two-phase ERP experiment revealed that reappraisal-based implementation intention produced an earlier and more sustainable emotion regulatory effects than controlled reappraisal.

During the first exposure task, we found that CR and RII reduced both the low- and high-intensity disgust experience effectively relative to the watching group. Consistent with previous findings, these behavioral findings suggest that automatic cognitive reappraisal (i.e., RII) as effective as controlled reappraisal (Shafir et al., 2015) in reducing both the low- and high-intensity adverse subjective experience. However, relative to the watching group, RII significantly reduced LPP amplitude across the entire time epoch (300–1,700 ms), whereas CR only reduced lower LPP amplitude within one time segment (500–700 ms). Furthermore, the neural regulatory effects of CR disappeared after corrections for multiple comparisons. We argue that the neural regulatory effects of RII have two advantages over that of CR. First, emotion regulation effects of RII on LPP amplitude were earlier than that of CR. In both low and high intensities, RII attenuated the centro-parietal LPP at the 300-ms picture presentation, whereas emotion regulation effects of CR started at 500 ms. Second, RII elicited a more sustainable attenuation of the centro-parietal LPP than CR in both low and high intensities. The modulating effect of RII lasted for a longer period (low/high, 300–1,700 ms) than CR (low/high, 500–700 ms), which supports our prediction that RII leads to a more sustainable emotion-regulatory effect than CR.

It may be argued whether RII participants in the first exposure task simply used attentional distraction as an effective method (i.e., “I will look away if I see a disgusting image”). However, the patterns observed for RII during the present re-exposure task significantly differed with those in the literature for attentional distraction. On the one hand, Thiruchselvam et al. (2011) found that upon unexpected re-exposure, pictures with a distraction (but not reappraisal) history elicited a larger LPP than images with a watching history and did not differ from pictures with a negative-watch history on self-reported ratings of valence and arousal. On the other hand, our unexpected re-exposure task showed that RII participants still self-reported lower ratings of emotional valence and arousal than CR and watching groups in both low and high intensities. In line with previous research (Thiruchselvam et al., 2011), we found no significant differences in subjective ratings between CR- and watching-history groups in both low and high intensities, confirming that the emotion-regulatory effects of CR on subjective experiences easily drop off with time. Moreover, the present results also demonstrated that only pictures with RII history (but not with CR history) elicited a lower amplitude of the centro-parietal LPP during the entire time window (300–1,700 ms). The subjective and ERP findings suggest that RII may integrate some advantages of distraction and reappraisal, as evidenced by that RII not only reduced the centro-parietal LPP amplitude earlier than CR during the first exposure task but also produced long-term emotion regulation effects during the unexpected re-exposure task.

Moreover, during the unexpected re-exposure task, results showed that RII also led to a lower amplitude of frontal LPP than the watching group, confirming the effortless characteristics of RII (Gallo and Gollwitzer, 2007; Gallo et al., 2009, 2012). To our surprise, we did not find a significant increase in frontal LPP amplitude during CR relative to watching groups, which is inconsistent with previous studies (Bernat et al., 2011; Moser et al., 2014; Shafir et al., 2015). A likely explanation is that previous studies of CR mainly used a within-participants design, requiring participants to switch between regulation and watch trials within one task (e.g., Moser et al., 2014). The trial of these studies commonly includes a blank or fixation (800 ms–1 s) between the cue and picture. In theory, participants have to retrieve information related to reappraisal into their explicit memory after viewing a cue of reappraisal. The memory preparation of reappraisal may be more effortful than that of simply passive watching. In contrast, the between-participants design only required participants to either passively watch pictures or reappraise their emotions during the entire task (e.g., Gallo et al., 2012). Participants did not need to switch their working memory back and forth between reappraisal and watching strategy frequently. Therefore, the between-participants design may be less effortful for participants to implement CR compared with the within-participants design of CR. In brief, the design to initiate CR, rather than CR itself, maybe cognitive costly (Richards, 2004).

Several limitations should be noted. First, the present study only focused on perspective-taking reappraisal and only used negative pictures involving blood to elicit disgust responses. These manipulations may result in the present task lacking ecological validity to understand the true ability of an individual to implement the automatic or controlled forms of cognitive reappraisal strategy. For example, it is unknown whether and how the results generalize to other forms of disgust, other negative emotions, and positive emotions. Second, the sample was entirely female, which may also limit the generalizability of our findings.

In summary, we first demonstrated that automatic reappraisal-based implementation intention yields an earlier and more sustainable emotion regulatory effects than controlled reappraisal, and such regulatory effects are not impacted by emotional intensity. These findings extend the process model of emotion regulation (Gross, 1998), suggesting that automatic and controlled forms of even one strategy have varying temporal trajectories.

## Data Availability Statement

The data that support the findings of this study are available on request from the corresponding author. The data are not publicly available due to privacy or ethical restrictions.

## Ethics Statement

The studies involving human participants were reviewed and approved by the local ethical committee of the Faculty of Psychology at Southwest University. The patients/participants provided their written informed consent to participate in this study.

## Author Contributions

SC, JYa, and JYu conceived and designed the experiments. SC and KY performed the experiments and analyzed the data. SC, JYa, and JYu wrote the article. All authors reviewed the manuscript.

## Conflict of Interest

The authors declare that the research was conducted in the absence of any commercial or financial relationships that could be construed as a potential conflict of interest. The handling editor is currently organizing a Research Topic with one of the authors JYu, and confirms the absence of any other collaboration.
