# Erratum: Automatic Reappraisal-Based Implementation Intention Produces Early and Sustainable Emotion Regulation Effects: Event-Related Potential Evidence

**DOI:** 10.3389/fnbeh.2020.594059

**Published:** 2020-08-21

**Authors:** Frontiers Production Office

**Affiliations:** Frontiers Media SA, Lausanne, Switzerland

**Keywords:** cognitive reappraisal, implementation intention, emotional intensity, event-related potentials, late positive potential

Due to a production error in the abstract, the last half of the sentence beginning with “Here, we aim to test” and the first half of the subsequent sentence were deleted. The text has been corrected to read as follows:

“Here, we aim to test whether automatic reappraisal-based implementation intention (RII) downregulates intense negative emotion more efficiently than controlled reappraisal (CR) using a two-phase event-related potential (ERP) experiment. In the regulation phase, both RII and CR decreased subjective experiences of negative emotion relative to passive watching, irrespective of emotional intensity.”

The publisher apologizes for this mistake. The original version of this article has been updated.

